# The District Nurse's Training

**Published:** 1909-06-12

**Authors:** 


### Nursing Administration.

### THE DISTRICT NURSE'S TRAINING.

The advocates of thorough training for district nurses had a hard battle to fight before they got their principles universally accepted. That the poor should have a lower class of nurse to the rich was never argued in crude form. But for a long time it was believed that the nurse who went in and out of the homes of the poor was unlikely to need the degree of skill demanded of her colleague in the sick-room, and might therefore assume her responsible duties without long preparation. The more far-sighted pioneers, among whose names that of Mrs. Dacre Craven deserves to be remembered, never wavered in the effort to make the public understand that the district nurse, liable continually to be thrown on her own resources, needs more rather than less training in comparison with the woman who works under the doctor's immediate supervision. The fixed period of training for district nurses rose from one year to two, and at last settled into the normal three years found necessary to ensure thorough competency in nurses of every description. Then came the discovery that three years' hospital training, although sufficient to impart a knowledge of nursing, does not fit a woman to enter at once upon district duties, and an additional six months spent in learning the details of district work in the homes of the poor is now considered essential.

It is undoubtedly a very important principle, in days when nurses drift from one branch of work to another at intervals of a few years, that the training of district nurses should in all essential particulars be identical with that of private nurses. The benevolent people who are always on the watch to persuade promising probationers to get a few months' training somewhere, in order to qualify for appointments at small wage, entirely forget the natural tendency of such imperfectly trained nurses to press forward and better themselves, not by adding to their qualifications, but by assuming duties for which they have never been prepared. Experience shows that few even of the nurses elaborately trained for the work of the district remain more than a few years in this groove, and that those who receive the minimum training through subsidies subscribed by village associations seldom continue village nurses after the termination of their original agreement. Whether they have received four years' or four months' training, the result is the same. They try some other kind of work—for a while, at any rate—often returning to district work when they have tasted variety.

Hence danger is incurred by bestowing a few months' training on uneducated women, and conferring on them the status of a nurse, while later on, supplied with a good testimonial, they are introduced to a sphere for which they are in no way fitted. So long as the district nurse is trained under precisely similar conditions to private nurses it matters little whether she varies her work, and in view of the fact that district work proves in the majority of cases only a temporary occupation, it is of the utmost importance that she shall not be trained exclusively with a view to this one kind of work.

It is unfortunate that hospital training can never suffice to fit women for district work without some supplementary experience. It discourages nurses from taking up district work to find that they are still probationers after they have received their certificates, and it is a heavy charge on charitable funds to be compelled to subsidise them while in the transition stage. At Guy's Hospital the selected nurses get an opportunity of taking a midwifery course, which lasts three months, and includes work in connection with the district nurses among the homes of the poor.

This is the ideal, but very few hospitals are able to offer their nurses this kind of experience. The nurse who takes up district work when launched out of the ward into a region wherein the merest necessaries are wanting is completely at a loss unless she is placed for a few months under the guidance of a superintendent who can minimise her difficulties and show her how a high standard of nursing can be maintained in the teeth of every obstacle. More training centres for this purpose are among the crying needs of the day. Not nearly all the available material is being used, and considering the high quality of the probationers who take up the work already fully qualified in other particulars, it ought not to be difficult to get them accepted as subordinate helpers in many parishes where now one fully qualified nurse is struggling single-handed with a condition of chronic overwork. No training is better than what can be imparted by a good teacher to one pupil working in close association with her. But it needs supplementing. Speakers at the recent Congress made a powerful plea for introducing some study of social conditions into the training of the district nurse.

District nursing is apt to narrow the intellect while developing the sympathies, because it is largely taken up with the performance of mechanical things not obviously related to the larger issues of life. The nurse's outlook becomes widened indefinitely when she obtains an insight into the trend of her daily small duties and perceives in what manner she is constituting a social force with far-reaching consequences for the race. She cannot know too much about the aims of the educationalist and of the sanitary expert, of the best traditions of charity and of the best aspirations of the new municipal reformers. She needs practical information about what is being done for the poor in her district, and how best to obtain for them benefits freely offered but often ignored. The vital need in the training of the district nurse is for some deeper instruction in the meaning of what she is attempting, so that she may be cheered with a sense of the value of her work. We are trying to get the best kind of nurses for this kind of work, and are succeeding only partially in retaining their services because nurses get tired of the day of small things. Are we, perhaps, training the fingers at the expense of the mind, and ought not the district nurse in continuation of thoroughly efficient training to be drawn more decidedly into the current of modern social movements?

